# Development of Novel High-Resolution Size-Guided Turbidimetry-Enabled Particle Purification Liquid Chromatography (PPLC): Extracellular Vesicles and Membraneless Condensates in Focus

**DOI:** 10.3390/ijms21155361

**Published:** 2020-07-28

**Authors:** Hussein Kaddour, Yuan Lyu, Nadia Shouman, Mahesh Mohan, Chioma M. Okeoma

**Affiliations:** 1 Department of Pharmacology, Stony Brook University Renaissance School of Medicine, Stony Brook, NY 11794-8651, USA; hussein.kaddour@stonybrook.edu (H.K.); yuan.lyu@stonybrook.edu (Y.L.); nadia.shouman@stonybrook.edu (N.S.); 2 Host Pathogen Interaction Program Southwest National Primate Research Center, Texas Biomedical Research Institute, San Antonio, TX 78227, USA; mmohan@txbiomed.org

**Keywords:** extracellular vesicles (EVs), exosomes, membraneless condensates (MCs), gradient size exclusion chromatography (gSEC), UV-Vis spectroscopy

## Abstract

Acellular particles (extracellular vesicles and membraneless condensates) have important research, drug discovery, and therapeutic implications. However, their isolation and retrieval have faced enormous challenges, impeding their use. Here, a novel size-guided particle purification liquid chromatography (PPLC) is integrated into a turbidimetry-enabled system for dye-free isolation, online characterization, and retrieval of intact acellular particles from biofluids. The chromatographic separation of particles from different biofluids—semen, blood, urine, milk, and cell culture supernatants—is achieved using a first-in-class gradient size exclusion column (gSEC). Purified particles are collected using a fraction collector. Online UV–Vis monitoring reveals biofluid-dependent particle spectral differences, with semen being the most complex. Turbidimetry provides the accurate physical characterization of seminal particle (Sp) lipid contents, sizes, and concentrations, validated by a nanoparticle tracking analysis, transmission electron microscopy, and naphthopyrene assay. Furthermore, different fractions of purified Sps contain distinct DNA, RNA species, and protein compositions. The integration of Sp physical and compositional properties identifies two archetypal membrane-encased seminal extracellular vesicles (SEV)—notably SEV large (SEV_L_), SEV small (SEV_S_), and a novel non-archetypal-membraneless Sps, herein named membraneless condensates (MCs). This study demonstrates a comprehensive yet affordable platform for isolating, collecting, and analyzing acellular particles to facilitate extracellular particle research and applications in drug delivery and therapeutics. Ongoing efforts focus on increased resolution by tailoring bead/column chemistry for each biofluid type.

## 1. Introduction

Most mammalian cells produce and release small acellular particles or structures into biofluids, including semen, blood, urine, milk, saliva, and conditioned culture medium (supernatant). These particles perform divergent physiological and pathophysiological functions depending on the cellular background that releases them. Two archetypes of acellular particle architectures are present in biofluids. One type is characterized by the presence of a lipid bilayer membrane, and a second type is characterized by the absence of a membrane. However, both archetypes have the presence of a bioactive cargo in common, i.e., the assemblage of proteins, nucleic acids, and potentially other macromolecules.

The most widely studied lipid bilayer membrane-encased particles are the nanosized extracellular vesicles (EVs). These membrane-enclosed nanoparticles are secreted by most cell types; are present in all body fluids, such as cerebrospinal fluid, urine, blood, saliva, breast milk, vaginal fluid, and semen, as well as cell culture supernatants; and these EVs facilitate distal and proximal intercellular communications [1,2]. Seminal studies in the past decade have demonstrated that EVs largely orchestrate recipient cell fates and may regulate disease pathogenesis [3], promote tumor progression [4], and regulate neurodegenerative disorders [5], among other roles. The diversity of EV-mediated regulations of cellular functions has been attributed to (i) their bioactive cargo, including different RNA species (mRNA and miRNA), proteins, lipids, and double stranded DNA (dsDNA) [6], and (ii) the ability to protect the cargo against degradation. These properties of EVs, as well as their endogenous natures, allow them to be considered as promising candidates of drug delivery and therapeutic agents [7].

The other acellular particle archetype comprises membraneless particles or membraneless condensates (MCs) that concentrate a wide array of bioactive molecules without an encapsulating membrane. According to in vitro studies, MCs assemble by thermodynamics-mediated liquid-liquid phase separation (LLPS) and aggregate biomolecules. MCs have been shown to regulate biological processes including, but not limited to, RNA metabolism, chromatin rearrangement, and signal transduction [8,9,10]. Noteworthy is that liquid MCs can transform into solid aggregates or reversible amyloid fibers. The amyloid fibers have been linked to the pathogenesis of amyotrophic lateral sclerosis [11,12], frontotemporal dementia [13], and even Alzheimer’s disease [14]. Thus, MCs may be functionally important in many disease processes, such as neurodegenerative diseases.

The isolation of EVs and MCs from biofluids requires stringent control to ensure the quality and production of particles that meet advanced analytical characterizations and preparative needs. Such needs require instrumentation with comprehensive online monitoring and retrieval capability, both of which will facilitate the control of critical process parameters, such as particle purity, the stratification into subpopulations, and the retrieval of preparative quantities. However, research on and the use of EVs and MCs have been largely hindered by technical difficulties related to the aforementioned parameters.

Currently, there are numerous methods available to purify EVs from body fluids and tissue culture samples [15] and none for MCs. These methods include differential ultracentrifugation, ultrafiltration, density gradient, flow cytometry, immunocapture, microfluidic isolation, size exclusion chromatography (SEC), and asymmetric-flow field-flow fractionation (AF4) [16,17,18,19,20]. None of these methods has been demonstrated efficient in subpopulation isolation that allows downstream functional analysis [15,21]. Recently, AF4 was optimized to permit the identification of membraneless particles called exomeres [22]. Though interesting, this technology has limitations, requires expensive instrumentation [23], and hence, is not broadly accessible. 

Here, we describe the development of a first-in-class high-resolution chromatographic size-guided turbidimetry-enabled dye-free system for the purification and analysis of EVs and MCs from biofluids, with semen as a model. This optimized method is based on the principle of size exclusion, where a novel column was carefully designed with a gradient-bead structure. This gradient size exclusion chromatography (gSEC) column, coupled with a small-volume fraction collector, allowed us to obtain an unprecedented high-resolution separation of particles into fractions of various subpopulations. UV-Vis spectroscopy, a widely accessible technique, was employed as a novel way to accurately identify the separated particles, calculate the particle size, and determine the particle concentration using turbidimetry. We validated the turbidimetry particle sizes and concentration measurements by transmission electron microscopy (TEM) and a nanoparticle tracking analysis (NTA), while the lipid concentration was validated by fluorescence spectroscopy. Immunoblotting, RNA profiling, and a proteomics analysis provided compositional validation. To our knowledge, this is the first report that successfully applied turbidimetry calculations to EV physical (size and concentration) characterizations. In summary, we have developed a simple but robust system with unmatched capability to separate biofluids into subpopulations of EVs and MCs in tandem with analytical and preparative features.

## 2. Results

### 2.1. Multi-Bead gSEC Column Isolate EVs from a Variety of Samples

**Figure 1:**
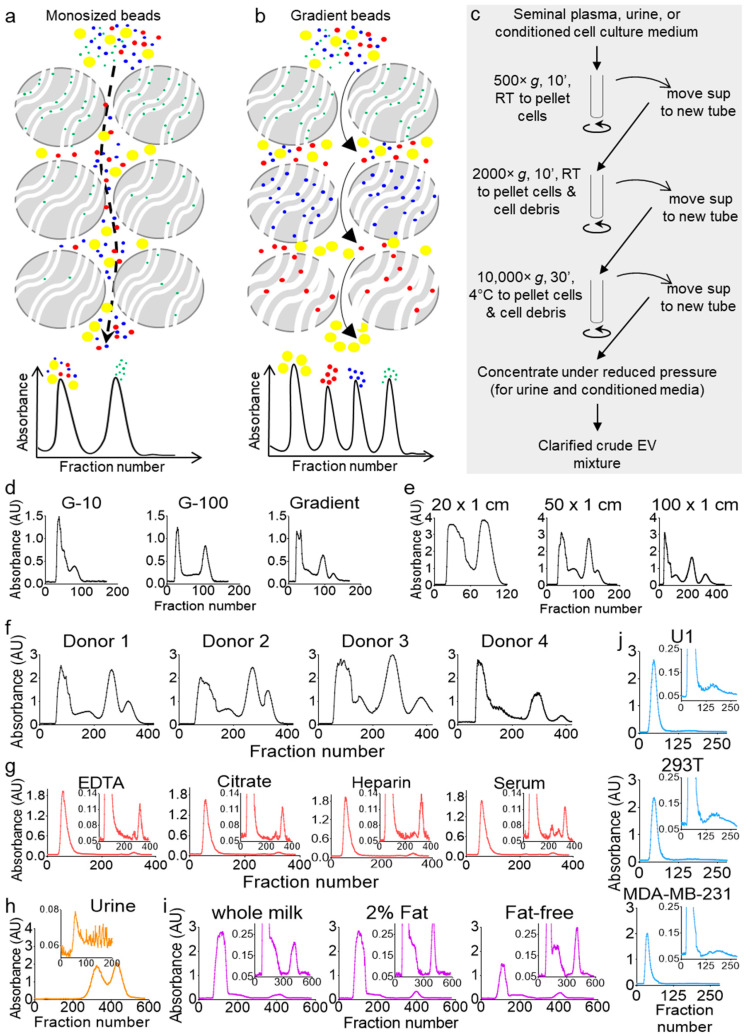
Columns packed with gradient size exclusion chromatography (gSEC) beads achieve higher resolution as compared to monosize bead columns. (**a**,**b**) Schematic describing that monosize bead separation (**a**) would end up with only two peaks, whereas separation with gSEC beads (**b**) may achieve a multi-peak resolution. (**c**) Schematic of the workflow for the clarification of crude extracellular vesicle (EV) mixtures from body fluids and cell culture supernatants by differential centrifugation before column separation. (**d**) Seminal plasma from 6 healthy men were pooled and clarified, and equal volumes (1 mL) were run on 50 cm × 0.5 cm G-10, G-100, or gSEC Sephadex bead-packed columns. Elution was carried out with phosphate-buffered saline (PBS), fractions were collected in 96-well plates, and the UV-Vis absorbance measurements were recorded using a plate reader. The profiles shown correspond to the 280-nm wavelength. (**e**) Gradient separation profiles of seminal plasma on columns of different lengths. Aliquots (1.5 mL) from the same pool of clarified seminal plasma (healthy, *n* = 6) were loaded on different-length Econo^®^ columns (20, 50, and 100 cm) packed with multi-size beads, with the elution and collection carried out under the same conditions. (**f**–**j**) Samples were purified on a 100 × 1 cm gSEC column. Fractions were eluted with PBS, collected in 96-well plates, and the UV-Vis absorbance measurements were recorded using a plate reader. (**f**) Seminal plasma separation profiles (1.5 mL) from 4 individual healthy donors. (**g**) Blood plasma and serum separation profiles. Blood was collected from 4 donors in different collection tubes, and clarified plasmas and serums were pooled by tube type. Three-hundred microliters of each pool were used for separation. (**h**) Forty milliliters of a first-void clean-catch urine sample was collected from a healthy male, clarified, and concentrated 10 times to 4 mL (Amicon™ ultra centrifugal filter unit, 3000 Da)—of which, 1.5 mL was used for purification. (**i**) Commercial cow milk separation profiles. Whole, 2% fat, and fat-free cow milk were purchased from Walmart, clarified by centrifugation at 10,000× *g* for 30 min, and 1 mL of each clarified sample was used. (**j**) U1, 293T, and MDA-MB-231 cells were cultured in 150 × 20 mm dishes in media supplemented with 10% exosome-depleted fetal bovine serum (FBS) for 3 days, and 10 mL of supernatants were clarified by differential centrifugation and concentrated (Amicon™ ultra-centrifugal filter unit, 3000 Da) to 1 mL before purification. All profiles shown correspond to the 280-nm wavelength. RT: room temperature.

Size exclusion separation is based on the principle of size discrimination, where larger molecules are excluded from the beads and flush-out directly, while smaller molecules are included in the beads and, hence, travel a longer time through the column. Thus, the large-sized molecules elute in the void peak, while the small-sized molecules elute in the latter peak (Figure 1a). Optimization of the separation parameters, such as the size and type of the beads and the length and width of the column, as well as the sample injection volume, can largely improve the separation profile by resolving the inclusion and exclusion peaks, but it cannot generate any additional peaks. Only a gradient of bead sizes allows the generation of additional peaks, where a particular sized molecule can be included in one bead size and excluded from the subsequent beads (Figure 1b). To test this idea, we clarified seminal plasma from a pool of six donors by differential centrifugation (Figure 1c) and separated equal aliquots on three Econo columns (50 cm × 0.5 cm) packed equally under atmospheric pressure with cross-linked dextran beads, Sephadex G-10; G-100; or a gradient of multi-beads (G-10, G-15, G-25, G-50, G-75, and G-100).

Four distinct peaks were obtained from the multi-bead gradient separation, while G-10 or G-100 monosize-bead columns allowed only a two-peak profile (Figure 1d). To validate the feasibility of our approach and evaluate the capability and flexibility of our column, we conducted additional studies. First, we tested different column sizes and, as expected, found that the sample resolution increased with an increase in the column length (Figure 1e). We then tested the separation of seminal plasma from four individual donors and found that the four-peak profile is donor-independent, albeit the ratios between the peaks were different (Figure 1f). We also separated blood plasma from a pool of four donors (Figure 1g) to find that, unlike seminal plasma, blood plasma consistently resolved into a three-peak profile, with the majority of the components present in the first peak, a very small second peak, and a sharp third peak. This profile was independent of the type of the blood-collection tube, although the no-additive blood serum profile showed a small fourth peak absent in the blood plasma profiles from the different anticoagulant-containing tubes. Interestingly, this difference between blood and seminal plasma may correlate to the differences in the blood and semen exosomal cargoes and functions [24,25]. We have indeed shown that blood and semen from the same donor contain distinct subsets of EVs that are compositionally and functionally different [24,26]. Furthermore, we profiled healthy human urine, which showed a unique three-peak profile with a very small first peak and large second and third peaks (Figure 1h). To evaluate the ability of the gSEC column to separate other biofluids, we used commercial pasteurized cow milk (one of the most wildly used nutritional biofluids) and a variety of cell culture supernatants (a widely used research biofluid). First, we used different grades (whole, 2% fat, and fat-free) of cow milk. Similar to seminal plasma, cow milk has a four-peak profile, with the majority of the components present in the first peak (Figure 1i). Although all four types of cow milk profiled with four peaks, subtle differences related to the amount of fat in the milk were observed (Figure 1i; compare the profiles of the insets). Next, we used cell culture supernatants. To this end, we cultured U1 cells (a U937-derived promonocytic cell line that are chronically infected with HIV-1), embryonic kidney epithelial (293T) cells, and metastatic breast cancer (MDA-MB-231) cells for three days in complete media supplemented with 10% EV-free fetal bovine serum (FBS). Ten millimeters of each supernatant were clarified, concentrated (Figure 1c), and then separated on gSEC. The cell culture supernatants showed two peaks (Figure 1j), and the absorbance profiles were different from those of the other biofluids, with subtle differences shown in the inset images. These results showed that multi-bead gSEC separation could be used to separate a variety of biological samples and that each biofluid (at least those tested here) has a unique characteristic absorbance profile. Since seminal plasma exhibit the most complex absorbance profiles and is of particular interest to our group [25,26,27,28,29,30], seminal plasma was used as a prototype biofluid for the characterization of the components in the peaks and the development of the analytical algorithms, as well as the compositional and functional studies.

### 2.2. Multi-Bead gSEC Column Isolates Different EV Subpopulations and MCs from Seminal Plasma

**Figure 2:**
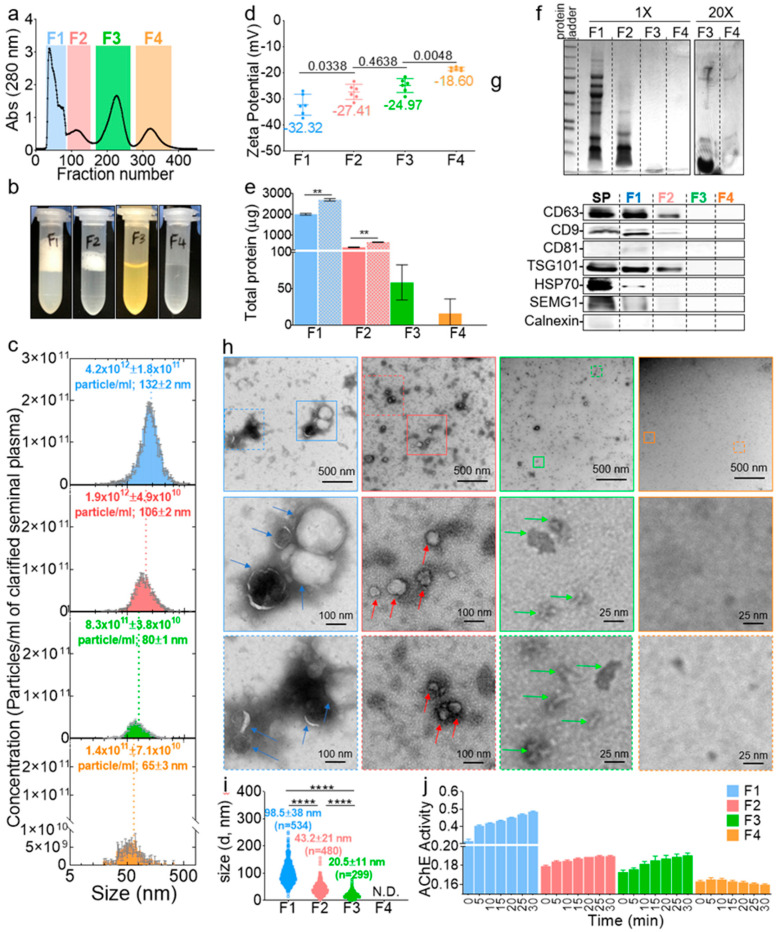
Human seminal plasma contains distinct EV subpopulations. (**a**) Collected fractions in 96-well plates were pooled into four fractions, frozen, and concentrated under reduced pressure, and the volume was adjusted to the input volume. To control for the amount of salts added to the samples, purification (which dilute the samples 10 times) was performed using 0.1× PBS buffer, and the volume of the concentrated samples was readjusted with ultra-pure water. Thus, the seminal plasma was separated into four fractions in 1× PBS buffer, without enriching or diluting the inherent components of each fraction. (**b**) Representative pictures of the four fractions after volume readjustment. (**c**) Size and concentration of F1–F4 by a nanoparticle tracking analysis (NTA). Error bars are standard deviation (SD) of triplicate measurements. Experiment was repeated at least three times, with similar results. (**d**) Zeta-potential as measured by NTA. Error bars are SD of pentaplicate measurements. Ordinary one-way ANOVA test (Tukey’s test) was used to determine the differences between F1–F4. Exact *p*-values are given in the figure. Experiment was repeated at least three times, with similar results. (**e**) Total protein in F1–F4 as quantified by the Bradford assay in the absence (filled bars) and presence (hatched bars) of triton X-100. Error bars are SD of duplicate measurements. Experiment was done once. Unpaired *t*-test with Welch’s correction was used to determine the differences between the groups. **, *p* < 0.01. (**f**) Representative sodium dodecyl sulfate polyacrylamide gel electrophoresis (SDS-PAGE), showing the protein profile of F1–F4. Experiment was repeated at least three times, with similar results. (**g**) Western blot of exosome markers. Loading was done by equal volume. SP, seminal plasma and SEMG1, seminogelin-1. (**h**) Representative negative-stain TEM images of F1–F4. Experiment was repeated at least three times, with similar results. (**i**) TEM-based mean particle size determined with Image J. At least three representative images from each of the three experiments were used for quantification. Ordinary one-way ANOVA test (Tukey’s test) was used to determine the differences between F1–F4. ****, *p* < 0.0001. N.D., not determined. (**j**) Acetylcholine esterase (AChE) enzymatic activity. Error bars are SD of triplicate wells. Experiment was repeated at least three times, with similar results.

Post-column fractions collected in 96-well plates were binned into four pool fractions (F), named F1–F4, frozen and concentrated under reduced pressure (Figure 2a). The volume of the fractions was then adjusted to the input volume; hence, the component’s concentration in each fraction was not different from the original seminal plasma concentration (Figure 2b). Interestingly, qualitative clues about the components of each fraction could be inferred visually where F1 and F2 had foamy top layers (more pronounced in F1), an indication of the presence of lipid vesicles [31], F3 was yellowish, indicating the potential presence of leftover urobilin from urine, since semen and urine both travel through the urethra, and F4 was clear, which indicated the presence of colorless components, such as fructose and minerals [32,33]. Of note, F3 always contained visible insoluble particulates after readjustment to the input volume. The NTA of F1–F4 validated the size-guided separation where the mean size of the vesicles decreased gradually (Figure 2c, *x*-axis). The concentration of the vesicles decreased as well where F1–F4 contained ~59%, ~27%, ~11%, and ~2% of the vesicles, respectively (Figure 2c, *y*-axis). The zeta potential was also different between the fractions, with a net negative surface charge decreasing from F1 to F4 (Figure 2d). The protein levels in the fractions were assayed in the presence and absence of triton. The results in Figure 2e show that the majority of the proteins (~80%) were in F1, and ~17%, ~2%, and ~1% of the proteins were distributed in F2–F4, respectively. The addition of triton significantly increased the levels of proteins in F1 and F2 but not in F3 or F4, confirming the presence of vesicles in F1 and F2, which lysed upon the triton treatment and released their protein cargo, whereas F3 and F4 may not contain vesicle-encased proteins. F1–F4 were then separated on a sodium dodecyl sulfate polyacrylamide gel electrophoresis (SDS-PAGE), which showed that, indeed, F1 and F2 contained most of the proteins, while F3, despite its high absorbance reading at 280 nm (Figure 2a), only contained small (~10 kDa) peptides, and F4 contained no detectable, even after loading 20 times more samples (Figure 2f). A Western blot analysis of known EV markers (CD9, CD81, CD63, HSP70, and TSG101) also pointed to the enrichment of EVs in F1 and F2 and their absence in F3 and F4 (Figure 2g). Next, negative stain TEM imaging (Figure 2h) was employed to further confirm the identity of the fractions. Structurally, F1 and F2 contain large and small membranous vesicles, respectively. Unlike these membranous vesicles, F3 is enriched in membraneless structures that are ~20 nm in size with defined sharp edges, while F4 contained neither vesicles nor any features. A quantitative analysis of the TEM images confirmed the enrichment of particles in F1, F2, and F3, in that order, with none in F4 (Figure 2i). Finally, we sought to access the level of a molecule known to be present both in EVs and outside of EVs as an additional validation of differences between the fractions. To this end, we accessed the acetylcholine esterase (AChE) level and activity. An AChE assay showed that all F1 contained most of the AChE activity, F2 and F3 contained residual activity, and F4 had no detectable activity (Figure 2j). This differential enrichment of AChE in the pooled fractions is consistent with a recent report [34] in which it was found that an AChE test, although convenient, cannot be used for EV detection, since acetylcholine may not be exclusive to EVs. Thus, given their size and presence or absence of a membrane, we named F1 large SEV (SEV_L_), F2 small SEV (SEV_S_), and F3 membraneless condensates (MCs). These results confirmed the presence and successful isolation of SEV_L_ and SEV_S_, as well as novel MCs from seminal plasma. These archetypal features of seminal vesicles may be connected to their functions.

### 2.3. UV-Vis Analysis Identifies the Molecular Components of the Purified Seminal Fractions

**Figure 3:**
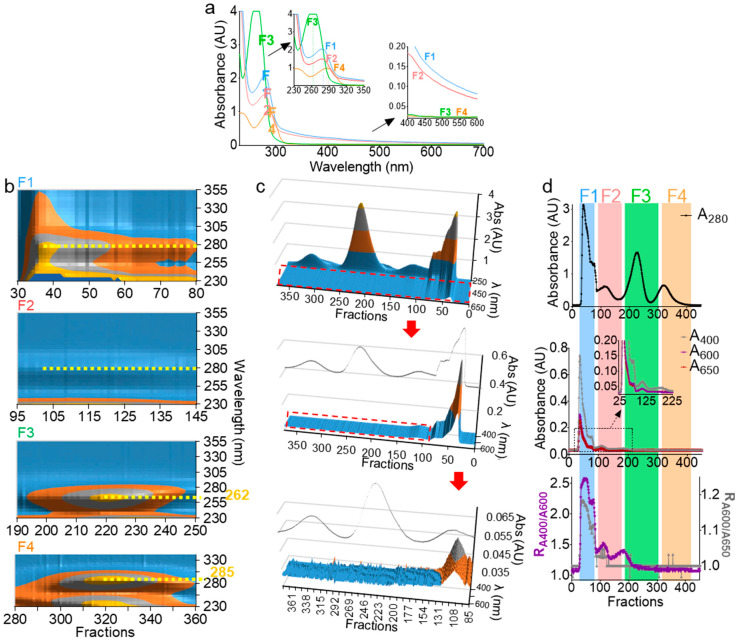
UV-Vis spectroscopy can accurately characterize EV subpopulations. (**a**) UV-Vis spectra of F1–F4. Top and bottom insets represent an enlarged graph of UV and visible spectra, respectively. Scans were performed from 230–700 nm with 2-nm intervals. (**b**) Contour view of 3D UV spectra of F1–F4 prior to pooling showing a 280-nm peak for F1 and F2, 262-nm peak for F3, and 285-nm peak for F4 (yellow dashed lines). (**c**) 3D surface plot of F1–F4 spectra, with a focus on the turbidity range showing the presence of a shoulder in F1 and F2 that is absent in F3 and F4, despite the high peaks in the UV range. The inset line corresponds to the 280-nm profile. Middle and bottom represent zoomed areas in the plot, indicated by a red dashed rectangle. (**b**,**c**) Plots were drawn in Microsoft Excel 2019. (**d**) A_280_ profile (top) as compared to A_400_, A_600_, and A_650_ profiles (middle). Bottom graph depicts R_1_ and R_2_ ratios (R_1_ = A_400_/A_600_, left axis, and R_2_ = A_600_/A_650_, right axis). Experiments were repeated at least three times, with similar profiles.

Absorbance at 280 nm (A_280_) has been used to determine the presence of EVs during size exclusion chromatography [35,36,37,38,39]; however, this wavelength is not ideal for EV detection, since free proteins may copurify with EVs. Monitoring EV separation in the turbidity range (400–600 nm) could be a better approach for EV detection. Indeed, the hydrophobic interlayer of the EV membrane scatters light in the visible spectrum range of light, making the lipid vesicle-containing solution turbid [40]. On the other hand, the UV spectrum range (190–350 nm) is essential, as it contains critical information regarding the nature, the concentration, and the purity of organic molecules. Thus, the full UV-Vis spectrum of F1–F4 was measured (Figure 3a). The shoulder in the turbidity range of F1 and F2 (Figure 3a) is indicative of the presence of membranous vesicles, while F3 and F4 are membraneless, given the absence of the turbidity range shoulder in the spectra (Figure 3a, bottom inset). In the UV range, F1 and F2 peaked at 280 nm, indicating protein decoration of the particle surfaces. Interestingly, F3 blue-shifted to ~262 nm, pointing to a potential presence of free nucleic acid, whereas F4, which contained the smallest molecules, red-shifted to ~285 nm, pointing to the presence of small peptides and minerals known to be present in seminal plasma [33,41,42]. This UV-Vis spectral analysis not only corroborated the results in Figure 2 and validated F1 and F2 as membrane-containing EVs fractions, but also presented new information in which MCs in the F3 fraction may be enriched in free nucleic acid aggregates. 

### 2.4. Three-Dimensional (3D) UV-Vis Profile Validates Components of the Purified Seminal Fractions

In order to test and extend the ranges of our system in depicting the nature of the biofluid-derived components, we employed 3D UV-Vis measurements (fraction/wavelength/intensity) in the UV range (Figure 3b) and visible range (Figure 3c). From this analysis, we identified with high-confidence vesicle-containing wells, which spanned over wells 29–125 (Figure 3c, red arrow and enlargement). Furthermore, in the visible range, we defined the ratios $R_{1}=A_{400}/A_{600}$ and $R_{2}=A_{600}/A_{650}$ as qualitative turbidity indices that are used concurrently to rule in or rule out the presence of vesicles (Figure 3d). The indices must be superimposed for a given well to contain vesicles. In contrast, if one index (often $R_{1}$) shows a peak that is absent in the other, it is an indication of the presence of impurities, such as colored materials. Indeed, in the example presented in Figure 3d, F1 and F2, which contain EVs, exhibit similar R_1_ and R_2_ profiles (Figure 3d, bottom), whereas F3, which lacks EVs, exhibited a peak only in R_1_, indicating the presence of a nonvesicular turbidity-exhibiting material (Figure 3d, bottom). Based on this analysis, it is now possible to quickly pinpoint vesicle-containing wells with high accuracy and without the need for full-spectra measurements. Nevertheless, 3D UV-Vis profiling during separation can be used as a simple yet powerful tool to identify a wide range of biomolecules in a sensitive and noninvasive manner.

### 2.5. UV-Vis Aanalysis Accurately Determines the Lipid Concentration of Purified EVs

**Figure 4:**
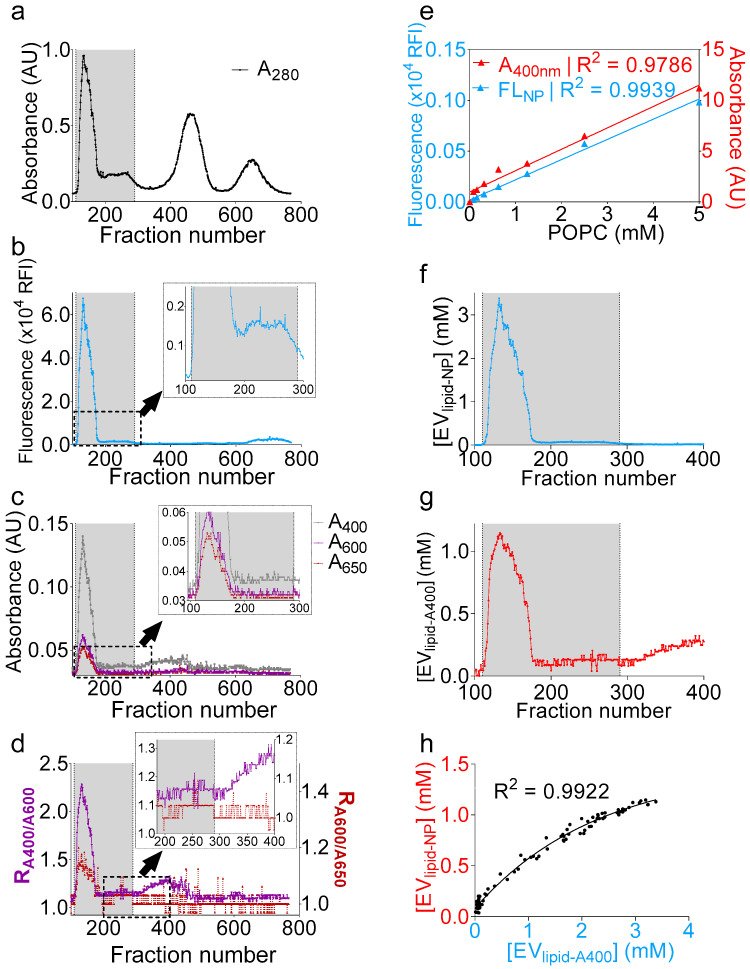
Naphtho[2,3-*α*]pyrene (NP) fluorescence and turbidity measurements accurately quantifies the total EV lipid concentration. One milliliter of clarified seminal plasma was incubated with naphthopyrene dye at a 1 µM final concentration, and, after brief tumbling at room temperature, the sample was purified on a gSEC column, and fractions were collected in 96-well plates (4 drops/well). (**a**–**c**) Separation profiles showing (**a**) A_280_; (**b**) NP fluorescence; and (**c**) A_400_, A_600_, and A_650_. Insets in **b** and **c** display zoomed area indicated by a dashed rectangle. The gray bar represents vesicle-containing fractions. (**d**) R_1_ (R_A400/A600_) and R_2_ (R_A600/A650_) ratios showing agreement over the EV range (100–290) and disagreement beyond fraction number 300. Inset display zoomed areas indicated by a dashed rectangle. (**e**) 1-palmitoyl-2-oleoyl-sn-glycero-3-phosphocholine (POPC) vesicles (5 mM) were prepared by the thin-film rehydration method in PBS, to which 1 µM naphthopyrene dye was added. A standard curve was prepared, and both A_400_ and NP fluorescence were recorded. Data were fitted to a simple linear regression using PRISM software. (**f**,**g**) Total lipid concentration inferred from the equations of the linear fit calculated in (**e**). (**f**), fluorescence and **g**, turbidity. (**h**) Nonlinear regression between the total lipid concentrations, as determined by turbidity and NP fluorescence, showing good agreement between both methods.

The successful qualitative determination of the presence of EVs encouraged us to deepen our analysis. We thus sought to convert turbidity into a quantitative parameter of an EV particle number. To this end, we added 1 µM of naphtho[2,3-*a*]pyrene (NP), a polycyclic aromatic hydrocarbon [43] that fluoresces only when embedded in the lipid bilayer [44,45], to the clarified seminal plasma. After a brief tumbling at room temperature using a rotary mixer, we purified the seminal plasma and monitored both the absorbance and fluorescence (Figure 4a–c) during the purification process. While raw turbidity profiles (A_400_, A_600_, and A_650_) will not rule in the presence of vesicles beyond the fraction number ~190 (Figure 4c), the NP fluorescence profile indicated that vesicle-containing wells extended until the fraction number ~290 (Figure 4c inset). On the other hand, R_1_ and R_2_ analysis revealed that fractions up to ~290 exhibited a peak slightly above the background noise, albeit small (Figure 4d and inset). Beyond the fraction number 290 (300–400), the increase of R_1_ without an increase in R_2_ indicated the presence of nonvesicular materials, an interpretation corroborating the lack of fluorescence in these fractions (300–400) (Figure 4d inset). The difference in the low detection range between the turbidity and fluorescence arises because the background in fluorescence is generally less pronounced compared to absorbance and because fluorescence is often more sensitive than absorbance, with a higher dynamic range. Nevertheless, it is possible to identify vesicle-containing wells using turbidity calculations. Subsequently, to attribute both turbidity and NP measurements to an absolute lipid concentration, we used a solution of known concentration of synthetic 1-palmitoyl-2-oleoyl-sn-glycero-3-phosphocholine (POPC) vesicles, to which NP was added at a concentration of 1 µM, and the mixture was serially diluted to generate a standard curve (Figure 4e). Both $A_{400}{}_{\mathrm{POPC}}$ and ${\mathrm{NP}}_{\mathrm{POPC}}$ strongly correlated to a linear regression (*R*^2^ = 0.9786 and 0.9939, respectively); thus, the total lipid concentration per fraction was inferred from the corresponding linear function for fluorescence (Figure 4f) and turbidity (Figure 4g). Although there was an underestimation in the concentrations determined by the turbidity method, as compared to the NP fluorescence, the regression between the data was strong, with a *R*^2^ of 0.9922 (Figure 4h). Finally, the data best correlated to an exponential fit that highlighted the differences in the dynamic ranges of detection between the two methods. Taken together, the data presented here not only show a novel and accurate way to determine the EV lipid concentration using NP fluorescence, but also suggest that monitoring turbidity during EV isolation could allow accurate and reproducible dye-free EV lipid quantifications. Note that the turbidity and NP analyses were not applied to MCs, because these fractions are membraneless.

### 2.6. UV-Vis Analysis Accurately Determines the Size and Particle Number of Purified EVs

**Figure 5:**
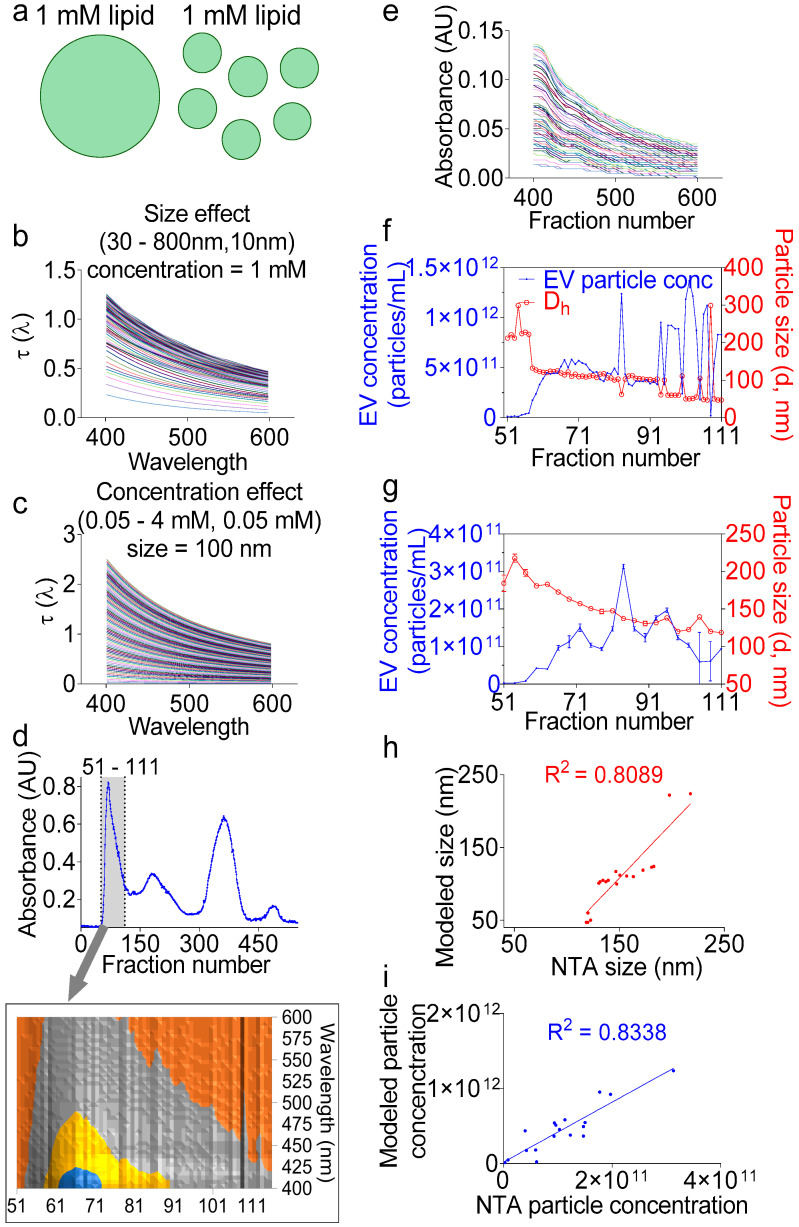
Turbidity measurements accurately determine the EV particle size and concentration. (**a**) Cartoon showing that the same total lipid concentration in a solution can form endless combinations of heterogeneous sizes and particle concentrations. (**b**,**c**) Representative modeled spectra for hypothetical hollow spheres with varying (**b**) lipid concentrations at a fixed particle size of 100 nm or (**c**) particle sizes at a fixed lipid concentration of 1 mM. (**d**) Separation profiles showing (A) 280-nm absorbance. Inset represents the 3D contour view of the F1 area, indicated by a gray lane (wells 51–111), and which was used in the subsequent calculations. (**e**) Measured turbidity spectra of the F1 area after removal of the background and transformation of the plate reader measured absorbance into turbidity. (**f**) EV particle concentration and hydrodynamic diameter (D_h_) as calculated from the measured turbidity spectra. (**g**) Particle size and concentration of individual fractions determined by NTA as an independent method of validation. (**h**,**i**) Linear regression for (**h**) size and (**i**) particle concentration between the turbidity model and NTA data showing good agreement between the two methods. Experiment was done at least twice, with similar goodness of the fit.

In the section above, we demonstrated the application of turbidimetry as a simple, effective, and dye-free way to accurately determine the lipid concentration in fractions during EV purification. However, the total lipid concentration alone does not permit particle number calculation without information about the particle size (Figure 5a). Fortunately, the UV-Vis measurements in our system are coupled with a high-resolution size-guided separation that yielded the populations of the monodisperse particles per fraction. This monodispersity implies that the particle size can be directly inferred from the turbidity spectra, whether by applying the exact Lorenz-Mie solution [40] or the Rayleigh-Gans-Debye approximation [46], without the need to invoke a distribution function, such as the log-normal Gaussian distribution [47]. Indeed, turbidity represents the attenuation of incident light due to light scattering and determination of the particle size spectrophotometrically. Turbidity measurements for size determination have been previously applied to nanoparticles [48], liposomes [46], and other lipid vesicles such as protocells [40], but not yet to EVs. Here, we applied the Lorenz-Mie turbidity model [40] to calculate the hypothetical turbidity spectra for the lipid vesicles of different concentrations and different sizes (Figure 5b,c), thus creating a matrix library of ~10,000 hypothetical spectra. We assumed in the library calculations an EV membrane thickness of 5 nm [49,50] and an EV lamellarity of 1 for biological membranes. We used for the medium and EV refractive indices the equations derived for water [51] and egg PC [52], respectively, as described previously [40]. We then calculated the turbidity spectra of F1 encompassing wells 51–111 (Figure 5d) by removing the background and multiplying the measured absorbance values by 2.303 (Equation (1), Figure 5e) [53]. Using the calculations from the above section, we determined the total lipid concentration per well, which was used to determine the EV particle size per fraction, as well as the EV particle number (Figure 5f). Indeed, we built on the Wang et al. model [40] and added additional command lines in which we aligned the measured spectra to the hypothetical spectra using the cost minimization function (Equation (2)), inputted the calculated lipid concentration per fraction, and calculated the particle number using Equation (3). To validate these calculations, NTA measurements were conducted on the individual fractions (Figure 5g), which also showed decreasing sizes, as expected from a size exclusion chromatography. As for the particle concentration, the NTA and turbidity calculations yielded similar overall numbers. The linear regression between the two methods for particle size and concentration determination showed good correlation with R squared of 0.8089 and 0.8335, respectively. A comparative analysis of the NTA vs. the turbidity size data show that the NTA sizes ranged from 217 to 118 nm, whereas the turbidity sizes spun over a wider range, from 224 to 47 nm (Figure 4e,g). It is thus tempting to say that the turbidity approach may be more suitable for EV size determination than the NTA. As for particle concentration, the turbidity calculation counted 3.06 × 10^13^ particles ~4.7-fold larger, as compared to the NTA volume-adjusted total particle number, which was 6.58 × 10^12^, indicating that the turbidity model may be more sensitive and suitable for measuring the EV particle number. In summary, we have presented a novel analytical method for the analysis of the EV size and concentration using the widely available turbidimetry assay without employing expensive NTA technology.

### 2.7. High-Resolution Chromatographic Size-Guided Turbidimetry-Enabled Dye-Free System Permits Identification of EV- and MC-Associated Cell-Free Nucleic Acids (cfNA)

**Figure 6:**
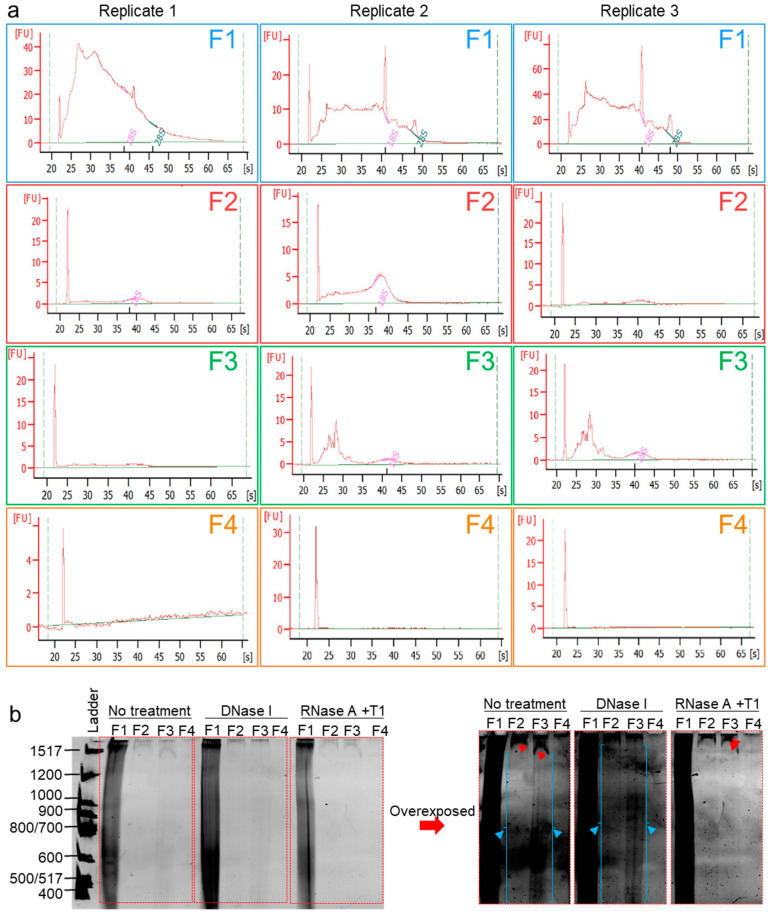
Cell-free nucleic acids (cfNA) are differentially enriched in EVs and EV-free seminal plasma. (**a**) RNA Bioanalyzer profiles of DNase I-treated RNA isolated from F1–F4. Experiments were repeated three times with different biological samples. (**b**) Denaturing PAGE of cfNA isolated by a phenol/chloroform extraction from F1–F4, untreated or treated with DNase I, or with a RNase cocktail (RNase A + RNase T1). Gel was run at 1000 V for 5 h before incubation in a Sybr^®^ Gold solution in the dark for 10 min and visualized under UV. Gel was imaged in normal exposure and overexposure settings. Red and blue arrows denote DNA and RNA bands, respectively. Experiment was repeated three times, with similar results.

It is known that human semen-derived EVs contain a repertoire of small noncoding RNA [54], and it is now evident that seminal RNA plays critical roles not only in sperm maturation and fertilization but also in embryo preimplantation and early embryogenesis [55,56,57]. Furthermore, human seminal EVs were demonstrated to contain DNA fragments ranging from ~500 to ~16,000 bp, but DNA (or RNA) species fractionation was not yet achieved [35,58]. We hypothesized that our separation method will separate different nucleic acid species. RNA was then extracted from F1–F4 using RNeasy^®^ with the optional on-column DNase I digestion performed, and eluted RNA samples were subjected to Agilent Bioanalyzer RNA profiling. The RNA profiles (Figure 6a) show decreasing sizes of RNA species with the separation, where large RNAs, including 18S and 28S rRNAs, are enriched in F1, and medium-sized RNA are enriched in F2, while small RNA (typical micro (mi)RNA-sized species) are enriched in F3, and F4 was RNA-free. In a separate experiment, the cfNA in F1–F4 were isolated by phenol/chloroform/isoamyl alcohol (25:24:1) at pH 8 and ethanol-precipitated. Purified cfNA were subjected to an 8% denaturing PAGE. The results show that F1–F4 are differentially enriched in cfNA, with F1 containing most of the cfNA, followed by F3, F2, and then F4 (Figure 6b). Furthermore, specific bands (indicated by red arrows, overexposed panel) disappeared upon RNase-free DNase treatment and remained in the RNase-treated lanes, demonstrating the cfDNA content of F2–F4. In contrast, the blue arrows indicated bands that persisted after DNase treatment and disappeared upon RNase treatment, pointing to the presence of RNA species in F2 and F3, as well. Finally, for F1, which contains over 90% cfNA, it is clear that it carries both RNA and DNA species. However, it was not possible to determine the enrichment of a species over the other, although it is tempting to say that the smear in F1 decreased more in the RNase than in the DNase treatment (Figure 6b, normal exposure), suggesting that F1 carries more RNA than DNA cargo. It is important to note that this gel does not show cfNA species smaller than 400 bp in length, and further experiments with a high-acrylamide percentage PAGE may be needed to assess the contents of small NA species, such as miRNA. Nonetheless, the bioanalyzer profiles (Figure 6a) demonstrate that our technique allows separation of the biofluids into fractions containing EV subpopulations or MCs prior to total RNA or DNA purification. This combination allows the fractionation of RNA species, which no other RNA isolation kit has achieved thus far.

### 2.8. High-Resolution Chromatographic Size-Guided Turbidimetry-Enabled Dye-Free System Permits Identification of EV- and MC-Associated Proteins

**Figure 7:**
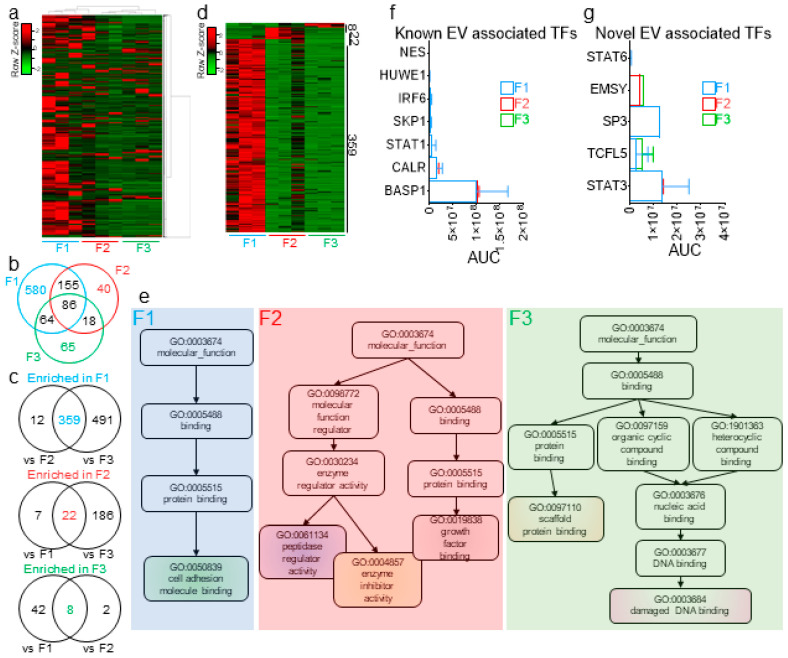
Extracellular proteins are differentially enriched in EVs and EV-free seminal plasma. (**a**) Cluster heatmap of the 2178 seminal plasma proteins identified in this study. (**b**) Venn diagram of the common and distinct proteins in F1–F3, as determined by the spectral count (SpC) method. (**c**) Venn diagram showing the common and distinct proteins significantly enriched in one fraction, as compared to the other two fractions. Significance of the differential enrichment was determined by two-way ANOVA using the area under the curve (AUC), as determined by the label-free quantification (LFQ) method, which was normalized prior to SpC. (**d**) Heatmap of the differentially enriched protein in each fraction. (**e**) Nonredundant Gene Ontology (GO) terms of the differentially enriched proteins in each fraction, as determined by WebGestalt analysis. (**f**,**g**) AUC of 7 previously identified (**f**) and novel cell-free (**g**) transcription factors, as quantified in each of the three fractions. Error bars represent the SEM of three biological samples. TF: transcription factors.

In prior studies, we used proteomics analyses to identify seminal proteins that are enriched in SEVs and those that are mostly present in EV-free seminal plasma [24]. Here, we conducted a MudPIT analysis of F1-3 from three biologically independent pools of seminal plasma. An F4 analysis was not performed, since in our separation profile and characterization of seminal plasma, F4 consistently contained no detectable proteins. The spectral count (SpC) data identified a total of 2178 proteins with at least one unique peptide (Figure 7a)—of which, 1204, 516, and 466 were common to all three biological replicates of F1, F2, and F3, respectively. Of those proteins, common and exclusive proteins were distributed among the three fractions (Figure 7b). To identify the enrichment pattern of the seminal plasma proteins in the fractions, we increased the stringency of our cutoff at the protein level to at least two unique peptides and performed ordinary two-way ANOVA tests between the different fractions and controlled for the false discovery rate (FDR), which was set to 0.05, using the original method of Benjamini and Hochberg. By these criteria, 359, 22, and 8 proteins were differentially present in F1, F2, and F3, respectively (Figure 7c,d). A nonredundant molecular function Gene Ontology (GO) analysis of these differentially present proteins revealed that F1 is enriched in proteins involved in cell adhesion molecule binding and F2 enriched in processes involving growth factor binding, enzyme inhibitor activity, and peptidase regulator activity, whereas F3 is enriched in proteins involved in scaffold protein binding and damaged DNA binding (Figure 7e). Furthermore, previously identified EV-associated transcription factors (TFs) [59,60], as well as five novel cell-free TFs (namely, STAT3, STAT6, TCFL5, EMSY, and SP3) were uncovered within the EVs and MCs (Figure 7f,g). This finding suggests that more regulatory proteins could be present with potential precise functions in the intended recipient cells and that further dissection of the cell-free seminal plasma fraction may facilitate their identification.

## 3. Discussion

Despite the increasing acknowledgment of the superiority of the size exclusion-based separation of EVs from biofluids [61], no EV subpopulation isolation has been achieved so far. Moreover, and importantly, it has been difficult to obtain preparative quantities of the EV subpopulation from any isolation method to date. We have developed and evaluated the utility of an optimized chromatography method based on a gradient of size exclusion multi-bead column that allows one-dimensional subpopulation EV isolation. We named this technology particle purification liquid chromatography (PPLC). PPLC was inspired from fast purification liquid chromatography (FPLC) and high-performance liquid chromatography (HPLC) systems, but unlike FPLC and HPLC, which are mainly used for large and small molecules, respectively, PPLC is designed with a focus on particles, such as EVs, viruses, liposomes, and synthetic nanocages. Regarding the column, the Sephadex beads have been introduced over sixty years ago [62], but, to our knowledge, this is the first time a gradient of beads was successfully used to achieve high-resolution separation of any particle type. Further, we innovatively coupled the gSEC column to a small-volume fraction collector, which allowed us to achieve an unprecedented high-resolution separation. Indeed, the drop-based fraction collector of PPLC allows the collection of as little as 22 µL per fraction (1 drop), rendering any sample to be fractionated into as many as ~3000 distinct fractions, based on the current column parameters where the elution between the void and the total volumes typically spanned over 500 fractions of six drops each. However, a simple scale-up—for instance, to a 170 × 1.5 cm column—would yield over 11,400 fractions. Thus, the PPLC versatility in scaling up coupled with the small fraction collection feature can achieve an unprecedented near-single-particle resolution.

Furthermore, and similarly to FPLC and HPLC, PPLC employs a UV-Vis detector, but unlike FPLCs and most of HPLCs, PPLC innovatively takes advantage of the full UV-Vis spectra in order to (i) accurately identify the fractions and (ii) determine the total lipid concentration, (iii) particle size, and (iv) particle concentration, as well as (v) assess the particle purity. The acquisition of these data currently requires expensive equipment such as flow cytometers, the NTA, and tunable resistive pulse sensing (TRPS) or reagents such as antibodies and colorimetric lipid quantification kits, which are based on the sulfo-phospho-vanillin colorimetric method [63,64]. However, and as demonstrated here, it is now possible to transform the easily acquired spectra of the widely-available UV-Vis spectroscopy into physiochemical information of prime biological importance. In addition, PPLC is superior to the NTA in many ways. First, the NTA recently failed to capture vesicle sizes smaller than 60 nm, and discrepancies in the reported particle concentrations were noted [65]. It is yet to independently evaluate the turbidity-based particle size and concentration determination; nevertheless, the results presented here point to a PPLC detection of 47–60 nm particles, whereas the NTA recorded 118–131 nm (Figure 5f,g—fractions 96–111). Second, the turbidity-based calculations do not require dilute samples, as opposed to the NTA, which only operates in a narrow concentration range of very diluted particles, making turbidity, but not the NTA, suitable for online tandem analytical and preparative systems such as PPLC. Finally, the NTA poorly differentiates EVs from protein aggregates. On the other hand, the 3D UV-VIS profiles of PPLC precisely distinguish EVs from protein aggregates and NA-rich components (Figure 3).

The research utility of PPLC cannot be over emphasized. It was recently shown that the major drawback in extracellular (ex)RNA studies is that different RNA isolation kits enrich different RNA species, making the reproducibility of RNA sequencing difficult to obtain [66]. PPLC solves this problem, because the data shown in Figure 6 demonstrate the feasibility of enriching for RNA species of interest during EV isolation. The data also show that MC-associated RNA can be queried using PPLC. To the best of our knowledge, no other RNA isolation kit can achieve this result. In addition to RNA, PPLC separates biofluids into fractions that contain distinct proteins/peptides, although some overlap is observed. So far, the best currently available EV subpopulation isolation technique is AF4 [22,23,67]. However, one of the limitations of AF4 is that the separation cell is very small, and thus scalability is not possible without extensive hardware modifications [23]. Furthermore, AF4 relies on a multi-angle light scattering detector for size calculations, which also requires dilute samples for measurements. Hence, scalability is difficult and, if to be attempted, must involve multiple runs of the same sample to obtain enough quantities for downstream functional studies [23]. In contrast, scalability in PPLC is easy, because it employs size exclusion [68], whose scalability has been demonstrated in bulk viral particles [69,70], as well as EV purifications [71]. Additionally, the UV-Vis detection in PPLC, unlike light scattering, is compatible with preparative separations, with a dynamic range of detection, from microabsorbance to absorbance units.

Above all, PPLC is capable of isolating, characterizing, and retrieving MCs that concentrate a wide array of bioactive molecules without an encapsulating membrane. Indeed, the copurification of EVs and MCs or other contaminants is an undesirable feature of most EV isolation protocols. PPLC solves this problem, as demonstrated in Figure 2. This feature cannot be overemphasized, because researchers can now separate EVs from MCs and avoid contaminants that oftentimes confound results in EV studies. Practically, it has been reported that cell-free proteins and nucleic acids copurify with EVs when other isolation methods, such as the miRCURY™ Exosome Isolation Kit, were used [72]. Precipitation-based EV isolation could coprecipitate lipoproteinw, 9–15% of plasma proteins, and 21–99% of vesicle-free miRNAs, as well as depending on the individual miRNA. Therefore, the PPLC solves this problem by separating EVs from MCs and other macromolecules—all from a single sample tube.

While PPLC as-is has surpassed the performances of rival isolation systems to date, there is room for improvement. Future optimization to the current PPLC is ongoing and includes column gradient ratio optimization to every biological sample, automation by the addition of a pump, and an autosampler for batch processing. A 2D separation profile with an anion exchange prepurification step is also under testing. We envisage building first-in-class PPLC for EV/MC isolation and characterization. Our goal is to offer two models: (i) analytical PPLC—expected to serve as a point-of-care test used to quickly check a patient’s EV or MC levels (UV-Vis profile, number, and size), which, when compared to a library of spectra, may give information about the physiological or pathological status of the patient. (ii) Preparative PPLC—suitable for EV or MC research, in which larger volumes are generally used, and fraction collection is available for downstream functional studies or utilization. Such a PPLC system could be the first one-box EV isolation and characterization device. Furthermore, such a device is expected to be affordable to most laboratories, since it will not require specific lasers for light scattering measurements and, hence, be universal.

Finally, the PPLC algorithm and the use of UV-Vis/turbidimetry calculations provides a real-time understanding of biological processes within biofluids that may allow the physiological status of the producer cells to be monitored continuously. In this backdrop, the archetypal features of PPLC-purified seminal particles may be connected to their function. Additional studies are required to functionally and mechanistically define SEV_L_, SEV_S_, and MCs.

## 4. Materials and Methods

### 4.1. Ethics

All experiments in this study were completed according to university regulations approved by The University of Iowa and Stony Brook University Institutional Review Boards (IRBs), with IRB numbers 201,608,703 and 2019-00507 for The University of Iowa and Stony Brook University, respectively. All participants were adults. They provided written informed consent for the semen samples, and all laboratory personnel were blinded to the clinical data. Cow milk was sourced commercially.

### 4.2. Samples Processing

Seminal specimens from healthy men collected by dry ejaculation were stored at −80 °C until used. The samples were thawed at room temperature (RT) and differentially centrifuged at 500× *g* for 10 min, 2000× *g* for 10 min, and 10,000× *g* for 30 min to remove the spermatozoa, leftover cells, and large materials, respectively. Samples were aliquoted either after pooling 3–6 samples or as individual donor aliquots and stored at −80 °C. Blood samples from 4 healthy donors were collected in different anticoagulant-type tubes (K_2_ EDTA, heparin, citrate, and no anticoagulant). The samples were left undisturbed for 2 h and then centrifuged at 2000× *g* for 10 min at RT. Serum and plasma were collected, centrifuged at 10,000× *g* for 30 min, and pooled by tube-type. Three-hundred microliters of each pool was used for separation. The rest of the samples were aliquoted and stored at −80 °C. Whole, 2% fat, and fat-free cow milk (Derle Hygrade) were purchased from Walmart, Selden, NY, USA. Twenty milliliters of milk samples, with at least 10 days prior to expiration, were centrifuged in 50 mL falcon tubes at 10,000× *g* for 30 min; the fat layer was carefully removed, and 1 mL was subjected to column separation. First-void clean-catch urine sample was collected from a healthy male and clarified by centrifugation at 2000× *g* for 10 min and 10,000× *g* for 30 min before concentration 10 times, from 40 mL to 4 mL, using an 3000 Da Amicon™ ultra centrifugal filter unit (Sigma-Aldrich, St. Louis, MO, USA), of which 1.5 mL was used for column separation. Cells, HIV-1 infected U1 (obtained through the NIH AIDS Reagent Program, Division of AIDS, NIAID, NIH from Dr. Thomas Folks [73]), 293T (ATCC), and MDA-MB-231 (ATCC) were cultured in 150 × 20 mm dishes for 3 days until confluency in complete media supplemented with 10% 18 h ultracentrifuged EV-depleted FBS (Atlanta Biologicals, Flowery Branch, GA, USA). Ten milliliters of each supernatant was clarified by differential centrifugation and concentrated (Pierce^TM^ Protein Concentrator 3K MWCO, Thermo Fisher Scientific, Waltham, MA, USA) to 1 mL and separated on a gSEC column.

### 4.3. Column Separation

All available size-exclusion dextran-based Sephadex^TM^ beads were purchased from Cytiva (formely GE Healthcare, Marlborough, MA, USA) and were as follows: G-10 (17-0010-01), G-15 (170020-01), G-25 fine (17-0032-01), G-50 fine (17-0042-01), G-75 (17-0050-01), and G-100 (17-0060-01). gSEC column was prepared by layering the beads from the smallest to the largest in different Econo-Columns^®^ (Bio-Rad, Hercules, CA, USA) at room temperature by gravity. The 1× or 0.1× phosphate-buffered saline (PBS) was used as the mobile phase. Fractions were collected in Greiner UV-Star^®^ 96-well plates using a FC204 fraction collector (Gilson, Middleton, WI, USA), with 6 drops per well. UV-Vis and fluorescence of the fractions were measured using a Synergy H1 plate reader (Biotek, Winooski, VT, USA).

### 4.4. Nanoparticle Tracking Analysis (NTA)

Size distribution and particle concentration of purified fractions were determined using ZetaView PMX110 (Particle Metrix, Mebane, NC, USA). The system was calibrated using 100 nm Nanosphere™ size standards (3100A, Thermo Fisher Scientific). Samples were diluted to the appropriate concentration in filtered ultrapure water, and measurements were acquired using ZetaView software v8.04.02. Shutter was kept at 70, and sensitivity was adjusted to 2–4 points below the noise level in an effort to capture the small particle. Measurements were taken in triplicates. For the zeta potential, samples were diluted in filtered PBS to the appropriate concentration for measurements, as noted by the software (usually between 80,000 to 200,000 times), and measurements were taken in pentaplicate. Experiments were repeated at least three times.

### 4.5. Acetylcholinesterase (AChE) Assay

Acetylcholinesterase enzymatic activity was measured as previously described [74]. Briefly, 15 µL of each fraction were lysed in 0.5% Triton X-100 in a 96-well plate, to which was added a solution of 100 µL of a 1:1 volumetric ratio of 1.25 mM acetylthiocholine chloride (Sigma-Aldrich, St. Louis, MO, USA) and 0.1 mM 5,5′-dithiobis2-nitrobenzoic acid (Sigma-Aldrich). Fifteen microliters of PBS was used as the AChE negative control. Absorbance was read at 450 nm for 30 min at 37 °C every 5 min on a plate reader (Synergy H1, Biotek). Data are reported as the mean from triplicate wells, and error bars are SD. Experiments were repeated at least three times.

### 4.6. SDS-PAGE Protein Profiles

One microliter from each fraction of the preparation was withdrawn for SDS–PAGE separation, which was carried out on 4–20% Bis-Tris gel (Bio-Rad, Hercules, CA, USA) for 120 min at 100 V. Gel was stained with Coomassie Blue. Since F3 and F4 contained low to no detectable levels of proteins, 20 µL of F3 and F4 were concentrated using a CentriVap benchtop vacuum concentrator (Labconco, Kansas City, MO, USA) and loaded in separate lanes. Experiment was repeated at least three times.

### 4.7. Western Blot

Primary antibodies against CD63, CD9 (mouse, Developmental Studies Hybridoma Bank, DSHB, Iowa City, IA, USA), CD81 (mouse, Proteintech, Rosemont, IL, USA), TSG 101 (rabbit, Proteintech, Rosemont, IL, USA), HSP70 (rabbit, R&D systems, Minneapolis, MN, USA), and Semenogelin-1 (SEMG-1, mouse, Santa Cruz Biotechnology, Dallas, TX, USA) were used for Western blot analysis. After incubation with primary and secondary IRDye 800CW donkey anti-mouse/rabbit IgG antibodies (LI-COR, Lincoln, NE, USA), the membranes were imaged with the LI-COR Odyssey Infrared Imaging System.

### 4.8. Transmission Electron Microscopy (TEM)

Transmission electron microscopy analysis of the isolated fractions was conducted as previously described [30]. Briefly, carbon-coated copper grids were glow-discharged to make the film hydrophilic (Pellco Easiglow, 0.2 mpar, 30 mA, 40 s, negative); then, ten microliters of F1–4 were applied to the grid and allowed to sit for 30 s. After removing the excess samples with filter paper, the grids were washed with distilled deionized water (ddH_2_O) twice, followed by staining with 0.7% uranyl formate solution for 20 s. The grids were allowed to air dry before viewed. TEI Tecnai12 BioTwinG 2 electron microscope was employed to view the samples, and an AMT XR-60 CCD digital camera system was used to capture the samples. Experiment was repeated three times. At least two images from each repeat were used in the particle size determination using ImageJ (NIH, Bethesda, MD, USA).

### 4.9. Preparation of 1-Palmitoyl-2-Oleoyl-Sn-Glycero-3-Phosphocholine (POPC) and Oleic Acid (OA) Vesicles

The method was described elsewhere [45,75]. Briefly, 5 mM phospholipid solution was prepared by evaporating 150 µL of 25 mg.ml^−1^ of POPC in chloroform (Avanti Polar Lipids, Alabaster, AL, USA) under a stream of nitrogen in a glass vial. The POPC thin film was hydrated with 1 mL of 1× PBS and tumbled overnight at RT on a rotary mixer. For OA vesicles, 32 µL of OA (Sigma Aldrich, St. Louis, MO, USA) were dissolved in 1 mL of NaOH (0.1 M) to form a 100 mM OA micelle solution, of which 50 µL were added to 1 mL of 1× DPBS to form a solution of 5 mM OA vesicles. Vesicles were tumbled overnight at RT on a rotary mixer. POPC and OA vesicles were extruded through various sizes of polycarbonate membranes (50–1000 nm) using a mini-extruder (Avanti Polar Lipids) to form monodisperse unilamellar vesicles.

### 4.10. Naphthopyrene (NP) Assay for Total Lipid Concentration

Naphtho(2,3-α)pyrene (>98%, TCI America, Portland, OR, USA) was dissolved in DMSO at a stock concentration of 2.5 mM. Two microliters of stock NP was added to 1 mL of clarified seminal plasma for a final NP concentration of 5 µM, and the mixture was incubated on a rotary mixer at RT for 1 h before gSEC column separation. In parallel, 5 mM phospholipid solution (POPC) was serially diluted, and NP was added for a final amount of 5 µM, corresponding to 0.1 mol% of POPC. Fluorescence (exitation/emission, 292/465 nm) and absorbance at 280 nm, 400 nm, and 600 nm were recorded for both the seminal plasma and the POPC standard curve. The standard curves data were fitted to the linear function, to infer the total lipid content in the seminal plasma fractions.

### 4.11. Vesicle Size and Concentration Modeling

For core-shell structures such as vesicles, the scattering cross-section depends on set parameters whose equations were previously described [40,46]. For our application, we favored the exact Lorenz-Mie solution over the Rayleigh-Gans-Debye approximation for two main reasons: First, the Lorenz-Mie solution applies to wider ranges of sizes, which fits the heterogenic nature of EVs [15,76,77], whereas Rayleigh scattering is only applicable in a narrow range of sizes where the particle radii should be significantly smaller than the wavelength of the scattered light [78]. Second, the exact Lorenz-Mie solution is more favorable than Rayleigh scattering when studying charged particles [79], which is also the case of EVs that have been reported to be negatively charged [30,80,81]. Thus, we applied the Wang et al. model [40], which was developed to calculate the scattering cross-section of concentric vesicles with an arbitrary size, lipid concentration, membrane thickness, or number of layers. This model uses the open-source light-scattering package HoloPy [82,83,84,85] (holopy.readthedocs.io/) and is available on GitHub with an illustrative example (https://github.com/anna-wang/vesicle-turbidity). In our analyses, we first calculated the EV turbidity spectra corresponding to F1 wells from the absorbance measured in the visible range (400–600 nm) with a 5-nm step using the following Equation (1) [40]:

(1) $$Calc.Turbidity_{\left(400–600\right)}=2.303\times \left(Absorbance_{\left(400–600\right)}-BG\right)$$

where *Absorbance*_(400–600)_ corresponds to the spectra measured by the plate reader, and BG is the background absorbance, mainly resulting from the plastic interference of the 96-well plate.

Next, we input *Calc. Turbidity*_(400–600)_ for each well of F1 together with its corresponding total lipid concentration that was calculated in the above section, and, for each input concentration, we generated an array of *Modeled Turbidity*_(400–600)_ spectra with a wavelength step of 5 nm for vesicles of a size ranging from 40 to 300 nm with a 1-nm step. This size range was chosen to encompass EVs of all sizes.

Subsequently, we computed a cost function (*CF*_400–600_) as follows:

(2) $$CF_{400–600}=\overset{n}{\underset{i=0}{\sum }}\left|y_{i}-{\hat{y}}_{i}\right|$$

where *n* is the number of wells for which the data is input, *i* is the index of the well, *y_i_* is the calculated turbidity based on the experiment, and ${\hat{y}}_{i}$ is the array of modeled turbidity for the same concentration as *i*. When *CF*_(400–600)_ reached a minimum for a given *i*, the vesicle size for that *i* corresponded to that from the closest *Modeled Turbidity*_(400–600)_.

Finally, with the hydrodynamic radius for each well now known, we calculated the vesicle concentration (*N_C_*) using the following equation, which was derived elsewhere [53] for hollow spheres:

(3) $$N_{C}=\frac{\left[L\right]10^{-3}N_{A}A_{L}}{4\pi \left[R^{2}+{\left(R-l_{B}\right)}^{2}\right]}$$

where *L* represents the lipid concentration (in M^−1^), $N_{A}$ is the Avogadro number, *R* is the radius of the vesicles, *l_B_* represents the bilayer thickness (5 nm) [49,50], *l_W_* represents the thickness of the interlamellar aqueous phase (3 nm) [53], and *A_L_* denotes the area per lipid (0.627 nm^2^) [86].

### 4.12. RNA Bioanalyzer

Twenty microliters of each fraction were purified using the RNeasy kit (Qiagen, Germantown, MD, USA) with an on-column DNase I digestion step. RNA was eluted with 14 µL of water and analyzed with an Agilent 2100 Bioanalyzer on an RNA 6000 pico chip (Agilent Technologies, Santa Clara, CA, USA), according to the manufacturer’s instructions. Experiment was repeated with three different biological replicates and yielded similar results.

### 4.13. Nucleic Acids Denaturing PAGE

Five-hundred microliters of each fraction were used for nucleic acid extraction twice with phenol/chloroform/isoamyl alcohol (25:24:1), pH 8.0 (Thermo Fisher Scientific) and twice with chloroform. The aqueous phase was transferred to a new tube, and the nucleic acids were precipitated with 300 mM sodium acetate, pH 5.2 and 2.5 equivalent volume of absolute ethanol. After chilling for 1 h at −86 °C, precipitated nucleic acids were pelleted by centrifugation (19,000× *g*, 20 min, 4 °C), and the pellets were resuspended in 100 µL water. Eighteen microliters of each nucleic acid solution was mixed with 2 µL of 10× DNase I reaction buffer (New England Biolabs, Ipswich, MA, USA), to which the vehicle PBS, 2.5 units RNase A and 100 units RNase T1 (RNAse cocktail A+T1, Invitrogen), 1 unit DNase I (New England Biolabs), or RNase and DNase together were added, and the tubes were incubated for 1 h at 37 °C. After 1 h, 20 µL stop solution (50% formamide, 50 mM EDTA, 0.1% bromophenol, and 0.1% xylene cyanol) was added, and samples were subjected to a denaturing 8 M urea PAGE. The gel was run at a 1000 V constant for 5 h and then stained with Sybr^®^ Gold stain (Thermo Fisher Scientific) for 20 min and visualized by UV at 254 nm.

### 4.14. Proteomic Analysis

Three seminal plasma pools (6 donors each) were clarified, and 1.5 mL of each pool was purified on a 100 × 1 cm gSEC column. Fractions 1–3 were concentrated under reduced pressure and quantified by the Bradford assay. The sample preparation and the LC parameters were recently described in detail [24]. Briefly, 50 µg of protein were denatured in urea, reduced with tris(2-carboxyethyl)phosphine (TCEP), alkylated with iodoacetamide, and digested overnight with Trypsin Gold (Promega). Tryptic peptides were desalted, dried, and resuspended in 5 μL of 0.5% fatty acids (FA) before loading onto a 3-phase MudPIT column, as described previously [87]. Peak lists, protein identifications, and database searches were conducted using BSI PEAKS Studio search engine software version 8.5 (Bioinformatics Solutions Inc., Waterloo, ON, Canada). For label-free quantitation (LFQ), Q module of BSI PEAKS was employed.

### 4.15. Sequence Databases

The Swiss-Prot UniProt Human nonredundant database (up000005640), which consisted of 20,303 annotated human proteins, was used as the reference database (https://www.uniprot.org/uniprot). Known contaminants to be excluded were identified and removed using the common Repository of Adventitious Proteins (cRAP) database version 1.0, release 2012.01.01 (https://www.thegpm.org/crap/). A derived score from the peptide-spectrum match (PSM) *p*-value known as the BSI PEAKS peptide score (−10 lgP) was used for the significance score of detection for all peptide-spectrum search results. A PEAKS protein score (sum of the −10 lgP peptide scores) of ≥20 was used as the significance threshold for all database search results. For the label-free quantitation (LFQ), an additional threshold of extracted-ion chromatogram (XIC) area under the curve (AUC) of the 3 most abundant peptides of a protein to be >1 e5 [88]. FDR was set to 0.1% at the peptide-spectrum match (PSM) level.

### 4.16. Data Mining and Visualization

Kyoto Encyclopedia of Genes and Genomes (KEGG) pathways and GO terms were determined using WEB-based Gene SeT AnaLysis Toolkit 2019 (www.webgestalt.org) [89]. Clustering heatmaps were drawn using a heatmapper [90]. The clustering method used was the average linkage with Euclidean distance measurement applied to both rows and columns. Venn diagrams were obtained using Venny platform (v2.1, publically available at https://bioinfogp.cnb.csic.es/tools.html).

## 5. Conclusions

In summary, PPLC uniquely combines performance and flexibility in a comprehensive affordable platform for the EV•MC isolation, characterization, and retrieval of intact particle subpopulations in a convenient 96-well format for downstream utilization. Owing to the extraordinary combination of gSEC, fraction collection, and UV-Vis/turbidimetry calculations, we show, for the first time, that a PPLC-generated spectral library, in combination with physical characteristics and compositional properties, can be used to identify acellular particles. Specifically, two archetypal and one nonarchetypal seminal particles (Sps) were found, and differences in their RNA and protein compositions were identified. As EVs and MCs are key mediators of distal and proximal cellular interactions and functions in physiological and pathophysiological conditions, PPLC provides a powerful tool to achieve the goal of isolating and retrieving pure, intact subpopulations of EVs and MCs for research. Within the context of drug delivery and therapeutic applications, PPLC offers a practical and robust platform not only for the isolation and retrieval of preparative quantities of EVs and MCs but, also, for isolating subpopulations of interest, although there is more work required to provide a comprehensive compositional and mechanistic description of the observations discovered herein.

## 6. Patents

On behalf of H.K. and C.M.O., Stony Brook University filed a patent application covering some aspects of the PPLC platform. Issues related to intellectual properties will be managed by the Office of Technology Licensing and Industry Relations (OTLIR) at Stony Brook University, according to existing and standard policies.

## Abbreviations

AChE	acetylcholine esterase
AF4	asymmetric-flow field-flow fractionation
cfNA	cell free nucleic acid
EVs	extracellular vesicles
exRNA	extracellular RNA
FPLC	fast purification liquid chromatography
gSEC	gradient size exclusion chromatography
HPLC	high performance liquid chromatography
LFQ	label-free quantification
MCs	membraneless condensates
NP	naphtho[2,3-a]pyrene
NTA	nanoparticle tracking analysis
OA	oleic acid
PAGE	polyacrylamide gel electrophoresis
POPC	1-palmitoyl-2-oleoyl-sn-glycero-3-phosphocholine
PPLC	particle purification liquid chromatography
SD	standard deviation
SEV	semen extracellular vesicles
SEV_L_	large semen extracellular vesicles
SEV_S_	small semen extracellular vesicles
SP	seminal plasma
Sps	seminal particles
TEM	transmission electron microscopy
TRPS	tunable resistive pulse sensing

## References

[1] Thery C., Zitvogel L., Amigorena S. (2002). Exosomes: Composition, biogenesis and function. Nat. Rev. Immunol..

[2] Raposo G., Stoorvogel W. (2013). Extracellular vesicles: Exosomes, microvesicles, and friends. J. Cell. Biol..

[3] Yang C., Robbins P.D. (2011). The roles of tumor-derived exosomes in cancer pathogenesis. Clin. Dev. Immunol..

[4] Bobrie A., Théry C. (2013). Unraveling the physiological functions of exosome secretion by tumors. OncoImmunology.

[5] Soria F.N., Pampliega O., Bourdenx M., Meissner W.G., Bezard E., Dehay B. (2017). Exosomes, an unmasked culprit in neurodegenerative diseases. Front. Neurosci..

[6] Thakur B.K., Zhang H., Becker A., Matei I., Huang Y., Costa-Silva B., Zheng Y., Hoshino A., Brazier H., Xiang J. (2014). Double-stranded DNA in exosomes: A novel biomarker in cancer detection. Cell Res..

[7] Hu G., Yang L., Cai Y., Niu F., Mezzacappa F., Callen S., Fox H.S., Buch S. (2016). Emerging roles of extracellular vesicles in neurodegenerative disorders: Focus on HIV-associated neurological complications. Cell Death Dis..

[8] Boehning M., Dugast-Darzacq C., Rankovic M., Hansen A.S., Yu T., Marie-Nelly H., McSwiggen D.T., Kokic G., Dailey G.M., Cramer P. (2018). RNA polymerase II clustering through carboxy-terminal domain phase separation. Nat. Struct. Mol. Biol..

[9] Tatavosian R., Kent S., Brown K., Yao T., Duc H.N., Huynh T.N., Zhen C.Y., Ma B., Wang H., Ren X. (2019). Nuclear condensates of the Polycomb protein chromobox 2 (CBX2) assemble through phase separation. J. Biol. Chem..

[10] Zhang Q., Huang H., Zhang L., Wu R., Chung C.I., Zhang S.Q., Torra J., Schepis A., Coughlin S.R., Kornberg T.B. (2018). Visualizing dynamics of cell signaling in vivo with a phase separation-based kinase reporter. Mol. Cell.

[11] Gui X., Luo F., Li Y., Zhou H., Qin Z., Liu Z., Gu J., Xie M., Zhao K., Dai B. (2019). Structural basis for reversible amyloids of hnRNPA1 elucidates their role in stress granule assembly. Nat. Commun..

[12] Patel A.N., Sampson J.B. (2015). Cognitive profile of C9orf72 in frontotemporal dementia and amyotrophic lateral sclerosis. Curr. Neurol. Neurosci. Rep..

[13] Mann J.R., Gleixner A.M., Mauna J.C., Gomes E., DeChellis-Marks M.R., Needham P.G., Copley K.E., Hurtle B., Portz B., Pyles N.J. (2019). RNA binding antagonizes neurotoxic phase transitions of TDP-43. Neuron.

[14] Kostylev M.A., Tuttle M.D., Lee S., Klein L.E., Takahashi H., Cox T.O., Gunther E.C., Zilm K.W., Strittmatter S.M. (2018). Liquid and hydrogel phases of PrPC linked to conformation shifts and triggered by alzheimer’s amyloid-β oligomers. Mol. Cell.

[15] Willms E., Cabañas C., Mäger I., Wood M., Vader P. (2018). Extracellular vesicle heterogeneity: Subpopulations, isolation techniques and diverse functions in cancer progression. Front. Immunol..

[16] Bobrie A., Colombo M., Krumeich S., Raposo G., Théry C. (2012). Diverse subpopulations of vesicles secreted by different intracellular mechanisms are present in exosome preparations obtained by differential ultracentrifugation. J. Extracell. Vesicles.

[17] Konoshenko M.Y., Lekchnov E.A., Vlassov A.V., Laktionov P.P. (2018). Isolation of extracellular vesicles: General methodologies and latest trends. BioMed Res. Int..

[18] Tauro B.J., Greening D.W., Mathias R.A., Ji H., Mathivanan S., Scott A.M., Simpson R.J. (2012). Comparison of ultracentrifugation, density gradient separation, and immunoaffinity capture methods for isolating human colon cancer cell line LIM1863-derived exosomes. Methods.

[19] Livshits M.A., Khomyakova E., Evtushenko E.G., Lazarev V.N., Kulemin N.A., Semina S.E., Generozov E.V., Govorun V.M. (2015). Isolation of exosomes by differential centrifugation: Theoretical analysis of a commonly used protocol. Sci. Rep..

[20] Maeki M., Kimura N., Sato Y., Harashima H., Tokeshi M. (2018). Advances in microfluidics for lipid nanoparticles and extracellular vesicles and applications in drug delivery systems. Adv. Drug Deliv. Rev..

[21] Mathieu M., Martin-Jaular L., Lavieu G., Théry C. (2019). Specificities of secretion and uptake of exosomes and other extracellular vesicles for cell-to-cell communication. Nat. Cell Biol..

[22] Zhang H., Freitas D., Kim H.S., Fabijanic K., Li Z., Chen H., Mark M.T., Molina H., Martin A.B., Bojmar L. (2018). Identification of distinct nanoparticles and subsets of extracellular vesicles by asymmetric flow field-flow fractionation. Nat. Cell Biol..

[23] Zhang H., Lyden D. (2019). Asymmetric-flow field-flow fractionation technology for exomere and small extracellular vesicle separation and characterization. Nat. Protoc..

[24] Kaddour H., Lyu Y., Welch J.L., Paromov V., Mandape S.N., Sakhare S.S., Pandhare J., Stapleton J.T., Pratap S., Dash C. (2020). Proteomics profiling of autologous blood and semen exosomes from HIV-infected and uninfected individuals reveals compositional and functional variabilities. Mol. Cell. Proteom..

[25] Welch J.L., Kaddour H., Winchester L., Fletcher C.V., Stapleton J.T., Okeoma C.M. (2020). Semen extracellular vesicles from HIV-1–infected individuals inhibit HIV-1 replication in vitro, and extracellular vesicles carry antiretroviral drugs in vivo. JAIDS J. Acquir. Immune Defic. Syndr..

[26] Welch J.L., Kaddour H., Schlievert P.M., Stapleton J.T., Okeoma C.M. (2018). Semen exosomes promote transcriptional silencing of HIV-1 by disrupting NF-kB/Sp1/Tat circuitry. J. Virol..

[27] Madison M.N., Roller R.J., Okeoma C.M. (2014). Human semen contains exosomes with potent anti-HIV-1 activity. Retrovirology.

[28] Madison M.N., Jones P.H., Okeoma C.M. (2015). Exosomes in human semen restrict HIV-1 transmission by vaginal cells and block intravaginal replication of LP-BM5 murine AIDS virus complex. Virology.

[29] Welch J.L., Madison M.N., Margolick J.B., Galvin S., Gupta P., Martínez-Maza O., Dash C., Okeoma C.M. (2017). Effect of prolonged freezing of semen on exosome recovery and biologic activity. Sci. Rep..

[30] Lyu Y., Kaddour H., Kopcho S., Panzner T.D., Shouman N., Kim E.Y., Martinson J., McKay H., Martinez-Maza O., Margolick J.B. (2019). Human immunodeficiency virus (HIV) infection and use of illicit substances promote secretion of semen exosomes that enhance monocyte adhesion and induce actin reorganization and chemotactic migration. Cells.

[31] Wang C.H., Yeh C.K. (2013). Controlling the size distribution of lipid-coated bubbles via fluidity regulation. Ultrasound Med. Biol..

[32] Schoenfeld C., Amelar R.D., Dubin L., Numeroff M. (1979). Prolactin, fructose, and zinc levels found in human seminal plasma. Fertil. Steril..

[33] Barrier-Battut I., Delajarraud H., Legrand E., Bruyas J., Fieni F., Tainturier D., Thorin C., Pouliquen H. (2002). Calcium, magnesium, copper, and zinc in seminal plasma of fertile stallions, and their relationship with semen freezability. Theriogenology.

[34] Liao Z., Jaular L.M., Soueidi E., Jouve M., Muth D.C., Schøyen T.H., Seale T., Haughey N.J., Ostrowski M., Théry C. (2019). Acetylcholinesterase is not a generic marker of extracellular vesicles. J. Extracell. Vesicles.

[35] Ronquist G.K., Larsson A., Stavreus-Evers A., Ronquist G. (2012). Prostasomes are heterogeneous regarding size and appearance but affiliated to one DNA-containing exosome family. Prostate.

[36] Grasso L., Wyss R., Weidenauer L., Thampi A., Demurtas D., Prudent M., Lion N., Vogel H. (2015). Molecular screening of cancer-derived exosomes by surface plasmon resonance spectroscopy. Anal. Bioanal. Chem..

[37] Taylor D., Gercel-Taylor C. (2005). Tumour-derived exosomes and their role in cancer-associated T-cell signalling defects. Br. J. Cancer.

[38] Blans K., Hansen M.S., Sørensen L.V., Hvam M.L., Howard K.A., Möller A., Wiking L., Larsen L.B., Rasmussen J.T. (2017). Pellet-free isolation of human and bovine milk extracellular vesicles by size-exclusion chromatography. J. Extracell. Vesicles.

[39] Takov K., Yellon D.M., Davidson S.M. (2019). Comparison of small extracellular vesicles isolated from plasma by ultracentrifugation or size-exclusion chromatography: Yield, purity and functional potential. J. Extracell. Vesicles.

[40] Wang A., Miller C.C., Szostak J.W. (2019). Core-shell modeling of light scattering by vesicles: Effect of size, contents and lamellarity. Biophys. J..

[41] Tvrdá E., Sikeli P., Lukácová J., Massányi P., Lukác N. (2013). Mineral nutrients and male fertility. J. Microbiol. Biotechnol. Food Sci..

[42] Talluri T.R., Mal G., Ravi S.K. (2017). Biochemical components of seminal plasma and their correlation to the fresh seminal characteristics in Marwari stallions and poitou jacks. Vet. World.

[43] Kwon J., Hong J.P., Lee W., Noh S., Lee C., Lee S., Hong J.I. (2010). Naphtho [2, 3, a] pyrene as an efficient multifunctional organic semiconductor for organic solar cells, organic light-emitting diodes, and organic thin-film transistors. Organ. Electron..

[44] Chen I.A., Salehi-Ashtiani K., Szostak J.W. (2005). RNA catalysis in model protocell vesicles. J. Am. Chem. Soc..

[45] Sahai N., Kaddour H., Dalai P., Wang Z., Bass G., Gao M. (2017). Mineral surface chemistry and nanoparticle-aggregation control membrane self-assembly. Sci. Rep..

[46] Elsayed M.M., Cevc G. (2011). Turbidity spectroscopy for characterization of submicroscopic drug carriers, such as nanoparticles and lipid vesicles: Size determination. Pharm. Res..

[47] Zender C. (2008). Particle Size Distributions: Theory and Application to Aerosols, Clouds and Aoils.

[48] Khlebtsov B.N., Khanadeev V.A., Khlebtsov N.G. (2008). Determination of the size, concentration, and refractive index of silica nanoparticles from turbidity spectra. Langmuir.

[49] Huber T., Rajamoorthi K., Kurze V.F., Beyer K., Brown M.F. (2002). Structure of docosahexaenoic acid-containing phospholipid bilayers as studied by 2H NMR and molecular dynamics simulations. J. Am. Chem. Soc..

[50] Leftin A., Molugu T.R., Job C., Beyer K., Brown M.F. (2014). Area per lipid and cholesterol interactions in membranes from separated local-field 13C NMR spectroscopy. Biophys. J..

[51] Van Engen A.G., Diddams S.A., Clement T.S. (1998). Dispersion measurements of water with white-light interferometry. Appl. Opt..

[52] Khlebtsov B., Kovler L., Bogatyrev V., Khlebtsov N., Shchyogolev S.Y. (2003). Studies of phosphatidylcholine vesicles by spectroturbidimetric and dynamic light scattering methods. J. Quant. Spectrosc. Radiat. Transf..

[53] Matsuzaki K., Murase O., Sugishita K.I., Yoneyama S., Akada K.Y., Ueha M., Nakamura A., Kobayashi S. (2000). Optical characterization of liposomes by right angle light scattering and turbidity measurement. Biochim. Biophys. Acta BBA Biomembr..

[54] Vojtech L., Woo S., Hughes S., Levy C., Ballweber L., Sauteraud R.P., Strobl J., Westerberg K., Gottardo R., Tewari M. (2014). Exosomes in human semen carry a distinctive repertoire of small non-coding RNAs with potential regulatory functions. Nucleic Acids Res..

[55] Jodar M. (2019). Sperm and seminal plasma RNAs: What roles do they play beyond fertilization?. Reproduction.

[56] Pang T.Y., Short A.K., Bredy T.W., Hannan A.J. (2017). Transgenerational paternal transmission of acquired traits: Stress-induced modification of the sperm regulatory transcriptome and offspring phenotypes. Curr. Opin. Behav. Sci..

[57] Park K.H., Kim B.J., Kang J., Nam T.S., Lim J.M., Kim H.T., Park J.K., Kim Y.G., Chae S.W., Kim U.H. (2011). Ca2+ signaling tools acquired from prostasomes are required for progesterone-induced sperm motility. Sci. Signal..

[58] Ronquist K.G., Ronquist G., Carlsson L., Larsson A. (2009). Human prostasomes contain chromosomal DNA. Prostate.

[59] Zhou J., Benito-Martin A., Mighty J., Chang L., Ghoroghi S., Wu H., Wong M., Guariglia S., Baranov P., Young M. (2018). Author Correction: Retinal progenitor cells release extracellular vesicles containing developmental transcription factors, microRNA and membrane proteins. Sci. Rep..

[60] Ung T.H., Madsen H.J., Hellwinkel J.E., Lencioni A.M., Graner M.W. (2014). Exosome proteomics reveals transcriptional regulator proteins with potential to mediate downstream pathways. Cancer Sci..

[61] Monguió-Tortajada M., Gálvez-Montón C., Bayes-Genis A., Roura S., Borràs F.E. (2019). Extracellular vesicle isolation methods: Rising impact of size-exclusion chromatography. Cell. Mol. Life Sci..

[62] Porath J., Flodin P.E.R. (1959). Gel filtration: A method for desalting and group separation. Nature.

[63] Chabrol E., Charonnat R. (1937). Une nouvelle reaction pour l’etude des lipides l’oleidemie. Presse Méd..

[64] Frings C.S., Dunn R.T. (1970). A colorimetric method for determination of total serum lipids based on the sulfo-phospho-vanillin reaction. Am. J. Clin. Pathol..

[65] Bachurski D., Schuldner M., Nguyen P.H., Malz A., Reiners K.S., Grenzi P.C., Babatz F., Schauss A.C., Hansen H.P., Hallek M. (2019). Extracellular vesicle measurements with nanoparticle tracking analysis–An accuracy and repeatability comparison between NanoSight NS300 and ZetaView. J. Extracell. Vesicles.

[66] Srinivasan S., Yeri A., Cheah P.S., Chung A., Danielson K., De Hoff P., Filant J., Laurent C.D., Laurent L.D., Magee R. (2019). Small RNA sequencing across diverse biofluids identifies optimal methods for exRNA isolation. Cell.

[67] Zijlstra A., Di Vizio D. (2018). Size matters in nanoscale communication. Nat. Cell Biol..

[68] Li Z., Gu Y., Gu T. (1998). Mathematical modeling and scale-up of size-exclusion chromatography. Biochem. Eng. J..

[69] Kaludov N., Handelman B., Chiorini J.A. (2002). Scalable purification of adeno-associated virus type 2, 4, or 5 using ion-exchange chromatography. Hum. Gene Ther..

[70] Lagoutte P., Mignon C., Donnat S., Stadthagen G., Mast J., Sodoyer R., Lugari A., Werle B. (2016). Scalable chromatography-based purification of virus-like particle carrier for epitope based influenza A vaccine produced in Escherichia coli. J. Virol. Methods.

[71] Corso G., Mäger I., Lee Y., Görgens A., Bultema J., Giebel B., Wood M.J., Nordin J.Z., Andaloussi S.E. (2017). Reproducible and scalable purification of extracellular vesicles using combined bind-elute and size exclusion chromatography. Sci. Rep..

[72] Karttunen J., Heiskanen M., Navarro-Ferrandis V., Das Gupta S., Lipponen A., Puhakka N., Rilla K., Koistinen A., Pitkänen A. (2019). Precipitation-based extracellular vesicle isolation from rat plasma co-precipitate vesicle-free microRNAs. J. Extracell. Vesicles.

[73] Folks T.M., Justement J., Kinter A., Dinarello C.A., Fauci A.S. (1987). Cytokine-induced expression of HIV-1 in a chronically infected promonocyte cell line. Science.

[74] Madison M.N., Welch J.L., Okeoma C.M. (2017). Isolation of exosomes from semen for in vitro uptake and HIV-1 infection assays. Bio-Protocol.

[75] Lyu Y., Fitriyanti M., Narsimhan G. (2019). Nucleation and growth of pores in 1, 2-Dimyristoyl-sn-glycero-3-phosphocholine (DMPC)/cholesterol bilayer by antimicrobial peptides melittin, its mutants and cecropin P1. Coll. Surf. B. Biointerfaces.

[76] Kowal J., Arras G., Colombo M., Jouve M., Morath J.P., Primdal-Bengtson B., Dingli F., Loew D., Tkach M., Théry C. (2016). Proteomic comparison defines novel markers to characterize heterogeneous populations of extracellular vesicle subtypes. Proc. Nat. Acad. Sci. USA.

[77] Van Niel G., d’Angelo G., Raposo G. (2018). Shedding light on the cell biology of extracellular vesicles. Nat. Rev. Mol. Cell Biol..

[78] Cox A.J., DeWeerd A.J., Linden J. (2002). An experiment to measure Mie and Rayleigh total scattering cross sections. Am. J. Phys..

[79] Li X., Xie L., Zheng X. (2012). The comparison between the Mie theory and the Rayleigh approximation to calculate the EM scattering by partially charged sand. J. Quant. Spectrosc. Radiat. Transf..

[80] Williams C., Pazos R., Royo F., González E., Roura-Ferrer M., Martinez A., Gamiz J., Reichardt N.C., Falcón-Pérez J.M. (2019). Assessing the role of surface glycans of extracellular vesicles on cellular uptake. Sci. Rep..

[81] Matsumoto A., Takahashi Y., Nishikawa M., Sano K., Morishita M., Charoenviriyakul C., Saji H., Takakura Y. (2017). Role of phosphatidylserine-derived negative surface charges in the recognition and uptake of intravenously injected B16BL6-derived exosomes by macrophages. J. Pharm. Sci..

[82] Wang A., Dimiduk T.G., Fung J., Razavi S., Kretzschmar I., Chaudhary K., Manoharan V.N. (2014). Using the discrete dipole approximation and holographic microscopy to measure rotational dynamics of non-spherical colloidal particles. J. Quant. Spectrosc. Radiat. Transf..

[83] Dimiduk T.G., Manoharan V.N. (2016). Bayesian approach to analyzing holograms of colloidal particles. Opt. Express.

[84] Wang A., Garmann R.F., Manoharan V.N. (2016). Tracking, E. coli runs and tumbles with scattering solutions and digital holographic microscopy. Opt. Express.

[85] Barkley S., Dimiduk T., Fung J., Kaz D., Manoharan V.N., McGorty R., Perry R., Wang A. (2019). Holographic microscopy with Python and HoloPy. Comput. Sci. Eng..

[86] Kučerka N., Nieh M.P., Katsaras J. (2011). Fluid phase lipid areas and bilayer thicknesses of commonly used phosphatidylcholines as a function of temperature. Biochim. Biophys. Acta BBA Biomembr..

[87] Link A.J., Eng J., Schieltz D.M., Carmack E., Mize G.J., Morris D.R., Garvik B.M., Yates J.R. (1999). Direct analysis of protein complexes using mass spectrometry. Nat. Biotechnol..

[88] Zhang J., Xin L., Shan B., Chen W., Xie M., Yuen D., Zhang W., Zhang Z., Lajoie G.A., Ma B. (2012). PEAKS DB: De novo sequencing assisted database search for sensitive and accurate peptide identification. Mol. Cell. Proteom..

[89] Liao Y., Wang J., Jaehnig E.J., Shi Z., Zhang B. (2019). WebGestalt 2019: Gene set analysis toolkit with revamped UIs and APIs. Nucleic Acids Res..

[90] Babicki S., Arndt D., Marcu A., Liang Y., Grant J.R., Maciejewski A., Wishart D.S. (2016). Heatmapper: Web-enabled heat mapping for all. Nucleic Acids Res..

